# Supplementary material for: Rescue of dendritic cells from glycolysis inhibition improves cancer immunotherapy in mice
Source: Nat Commun. 2023 Sep 2;14:5333. doi: 10.1038/s41467-023-41016-z (PMC10475105; doi:10.1038/s41467-023-41016-z)
Supplement: Supplementary file 1 — Supplementary Information [file 41467_2023_41016_MOESM1_ESM.pdf]

Supporting Information

Rescue of dendritic cells from glycolysis inhibition improves cancer immunotherapy in mice

Sahil Inamdar[1,#], Abhirami P. Suresh[2,#], Joslyn L. Mangal[2], Nathan D. Ng[3], Alison Sundem[1], Christopher Wu[4], Kelly Lintecum[1], Abhirami Thumsi[2], Taravat Khodaei[4], Michelle Halim[1], Nicole Appel[1,5], Madhan Mohan Chandra Sekhar Jaggarapu[1], Arezoo Esrafili[1], Jordan R. Yaron[1], Marion Curtis[6,7], Abhinav P. Acharya[1,2,4,5,8,9,*]

[1]*Chemical Engineering, School for the Engineering of Matter, Transport, and Energy, Arizona State University, Tempe, AZ, 85281, USA*

[2]*Biological Design, Arizona State University, Tempe, AZ, 85281, USA*

[3]*Molecular Biosciences and Biotechnology, The College of Liberal Arts and Sciences, Arizona State University, Tempe, AZ, USA, 85281*

[4]*Department of Biomedical Engineering, School of Biological and Health System Engineering, Arizona State University, Tempe, AZ, 85281, USA*

[5]*Center for Immunotherapy, Vaccines and Virotherapy, Arizona State University, Tempe, AZ, 85281, USA*

[6]*Department of Cancer Biology, Mayo Clinic, Scottsdale, AZ, USA, 85259 8*

[7]*College of Medicine and Science, Mayo Clinic, Scottsdale, AZ, USA, 85259,*

[8]*Materials Science and Engineering, School for the Engineering of Matter, Transport, and Energy, Arizona State University, Tempe, AZ, 85281, USA*

[9]*Biodesign Center for Biomaterials Innovation and Translation, Arizona State University, Tempe, AZ, 85281, USA*

* Corresponding author - email - abhi.acharya@asu.edu # These authors contributed equally

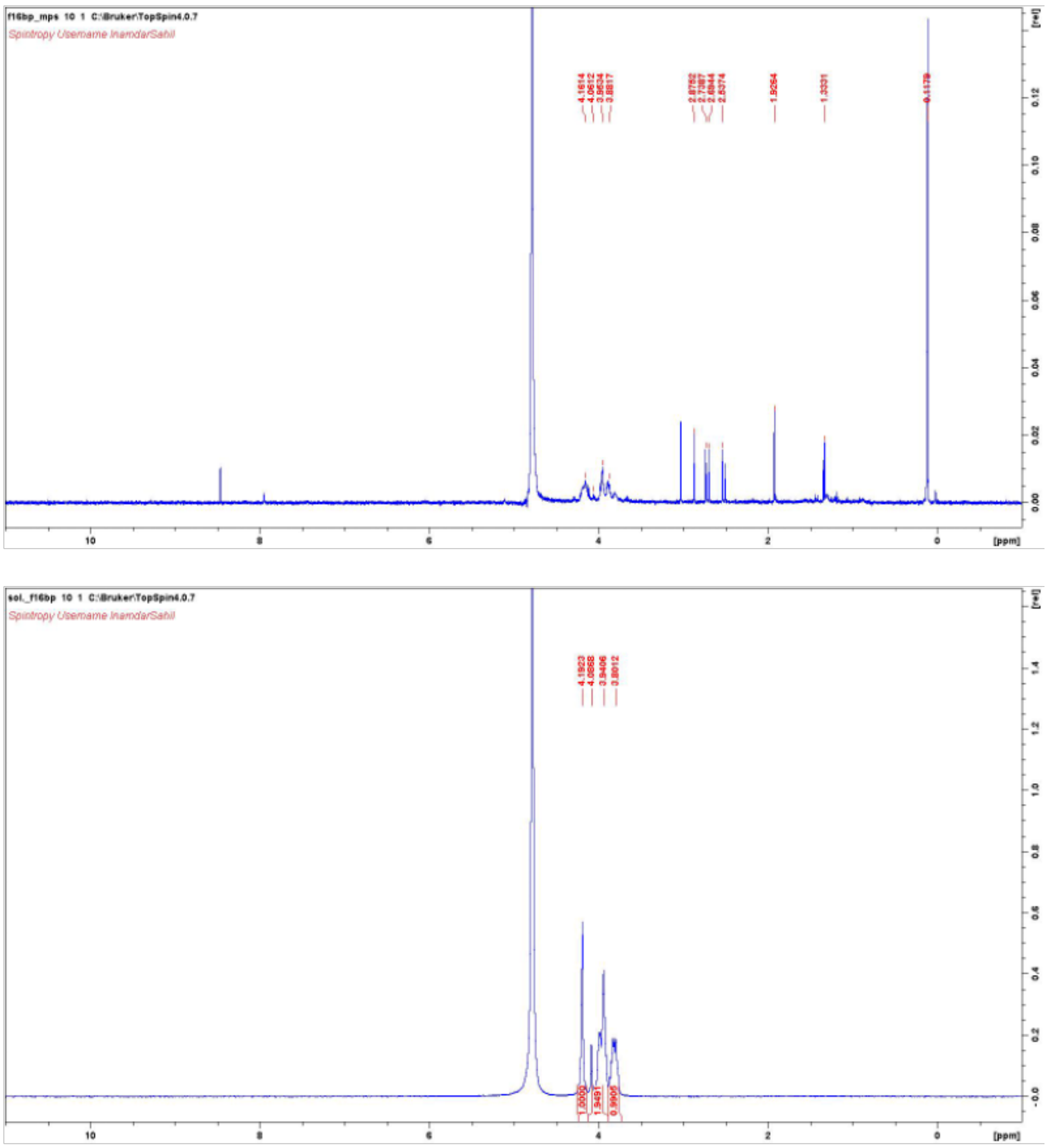

Figure S1: Nuclear Magnetic Resonance (NMR) indicates the presence of F16BP in the F16BP MPs. 1H NMR (500 MHz, D2O): δ 4.981(d, 1H), δ 4.1923 (s, 1H), δ 4.0868 (s, 1H), δ 3.9406 (dd, 1H), δ 3.8012 (d, 1H).

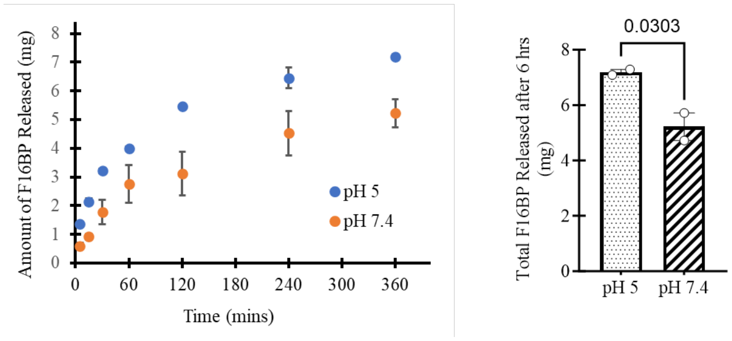

Figure S2: F16BP MPs could release F16BP for 6 hours in a sustained manner. Data represented as mean ± std err.

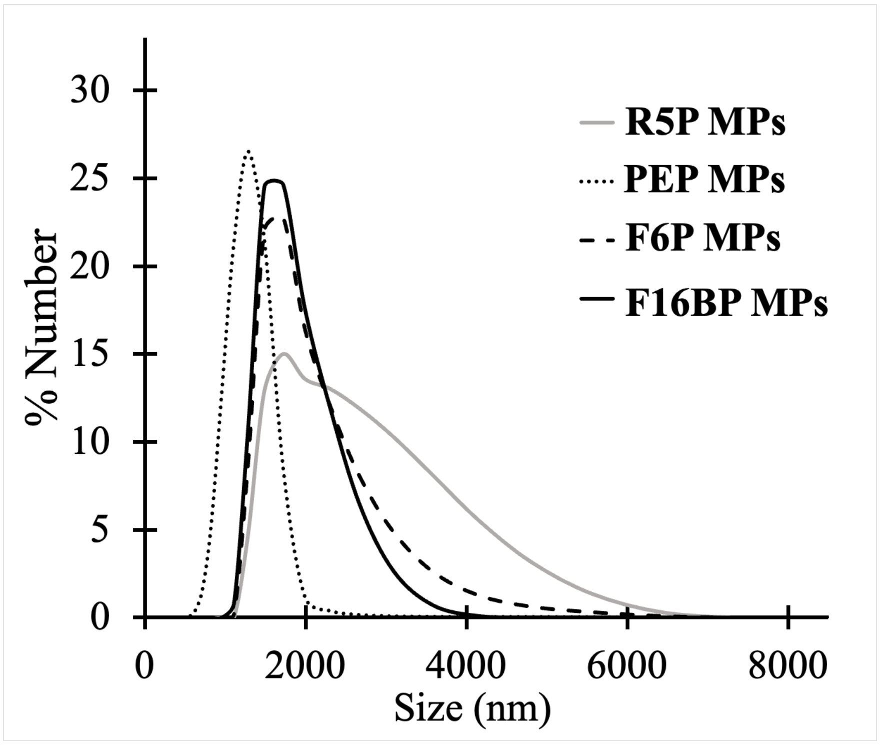

	R5P	PEP	F6P	F16BP
Average (nm)	3576	2247	3179	2343
Std Dev (nm)	408	205	519	363

Figure S3: Microparticles of other control metabolites such as ribulose 5 phosphate (R5P), Phosphoenolpyruvic acid, and fructose 6 phosphate (F6P) were generated.

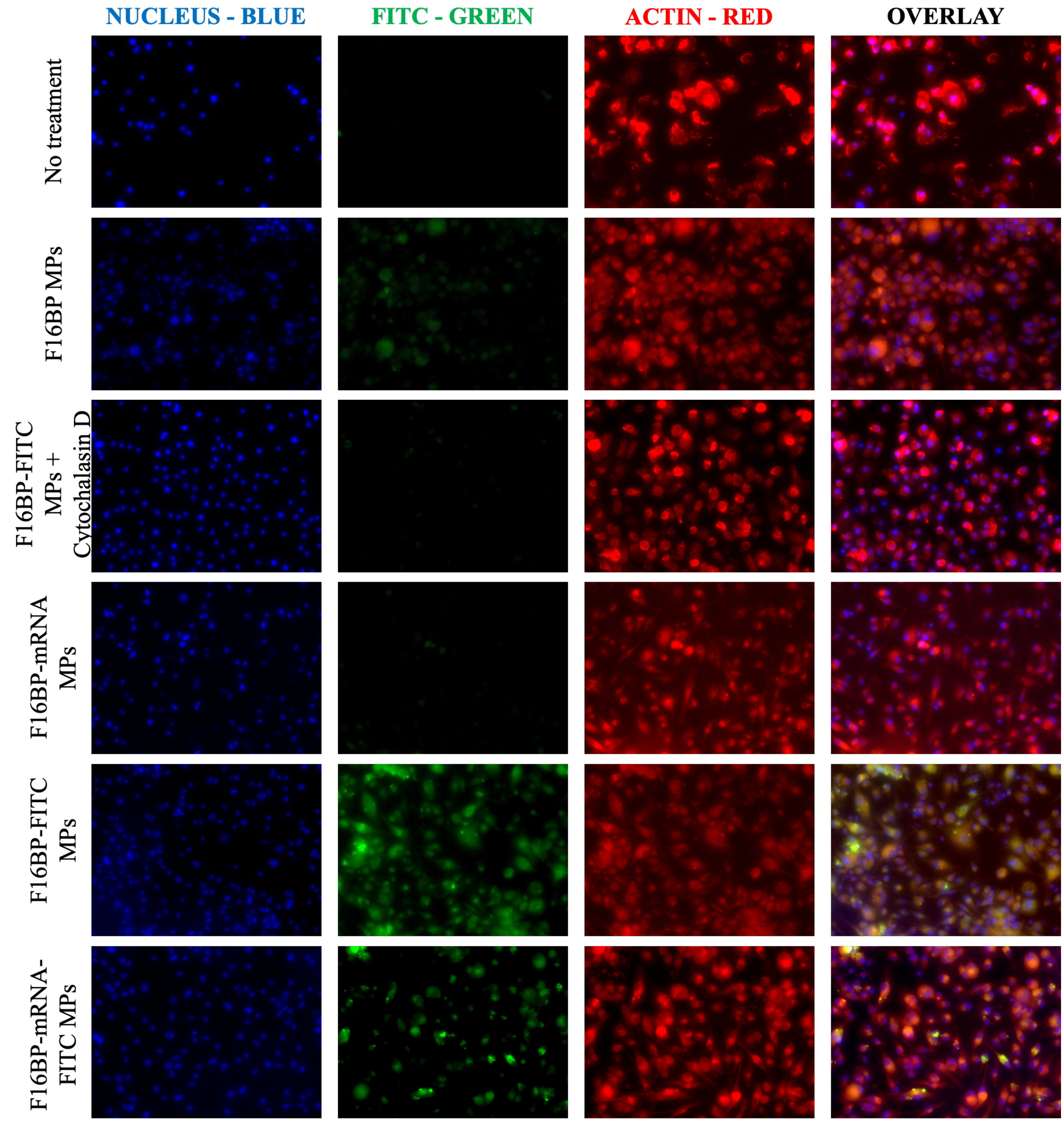
NUCLEUS - BLUE
FITC - GREEN
ACTIN - RED
OVERLAY
No treatment
F16BP MPs
F16BP-FITC MPs + Cytochalasin D
F16BP-mRNA MPs
F16BP-FITC MPs
F16BP-mRNA-FITC MPs

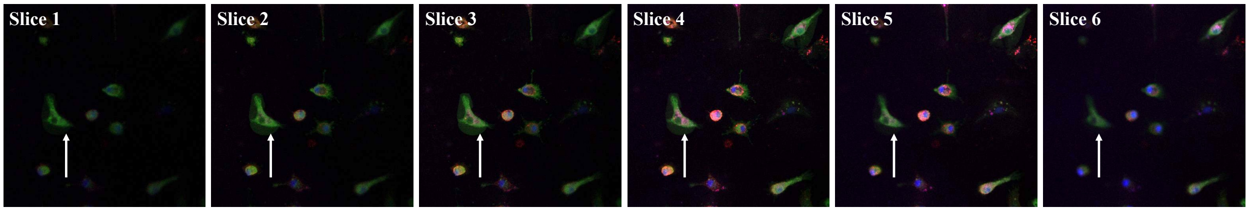
Slice 1
Slice 2
Slice 3
Slice 4
Slice 5
Slice 6

Figure S4: Confocal microscopy image indicates the phagocytosis of F16BP MPs by DCs. (Top panel) Confocal representative images of different conditions. **(Bottom panel)** F16BP-mRNA-FITC MPs, slices at different z-axis demonstrates that the particles (pseudo color RED), are intracellularly present in dendritic cells (pseudo color actin – green; nucleus – blue) Scale bar = 100 μm.

Table 1 - Following are the reagents and antibodies used for Flow cytometry in the study:

	Target	Fluorophore	Company	Catalog #	Clone
1	CD4	PE	BD	12-0041-82	GK1.5
2	CD8	APC-R700	BD	564983	53-6.7
3	CD25	PECy7	BD	552880	PC61
4	CD11c	PE	BioLegend	117308	N418
5	CD86	SB600	Thermo	63-0862-82	GL1
6	CD80	PE-Cy5	Invitrogen	15-0801-82	16-10A1
7	MHCII	APC	BioLegend	107614	M5/114.15.2
8	Tbet	BV785	BioLegend	644835	4B10
9	FoxP3	eF450	Invitrogen	48-5773-82	FJK-16s
10	RORγT	BV650	BD	564722	Q31-378
11	Ki67	FITC	Invitrogen	11-5698-82	SolA15
12	GATA3	BV711	BD	565449	L50-823
13	CD16/CD32:Fc Block	NA	Tonbo	70-0161-M001	2.4G2
14	F4/80	BV702	Invitrogen	67-4801-80	BM8
15	Comp beads	NA	Invitrogen	01-2222-42	NA
16	Cell stimulation cocktail (with golgi stop)	NA	Tonbo	TNB-4975-UL100	NA

17	Cell activating cocktail (w/o brefeldin A)	NA	BioLegend	423301	NA
18	L/D	eF780	NA	NA	NA
19	IL12	V450	BD	561456	C15.6
20	IFNY	PE	Tonbo	50-7311-U100	XMG1.2
21	IL10	PE/DAZZLE	BioLegend	505034	JES5-16E3
22	TNFa	BV510	BD	563386	MP6-XT22
23	Golgi Stop	NA	BD	554724	NA
24	Golgi Plug	NA	BD	555029	NA
25	CD11b	FITC	Tonbo	35-0112-U500	M1/70
26	CD3	NA	BioLegend	100202	17A2
27	CD28	NA	Tonbo	70-0281-U100	37.51
28	CD163	SB436	ThermoFisher	62-1631-82	TNKUPJ
29	CD206	PECy7	ThermoFisher	25-2061-82	MR6F3
30	Foxp3 / Transcription Factor Staining Buffer Set (Fix/Perm concentrate and dilutent, and 10x perm buffer)	NA	ThermoFisher	00-5523-00	NA

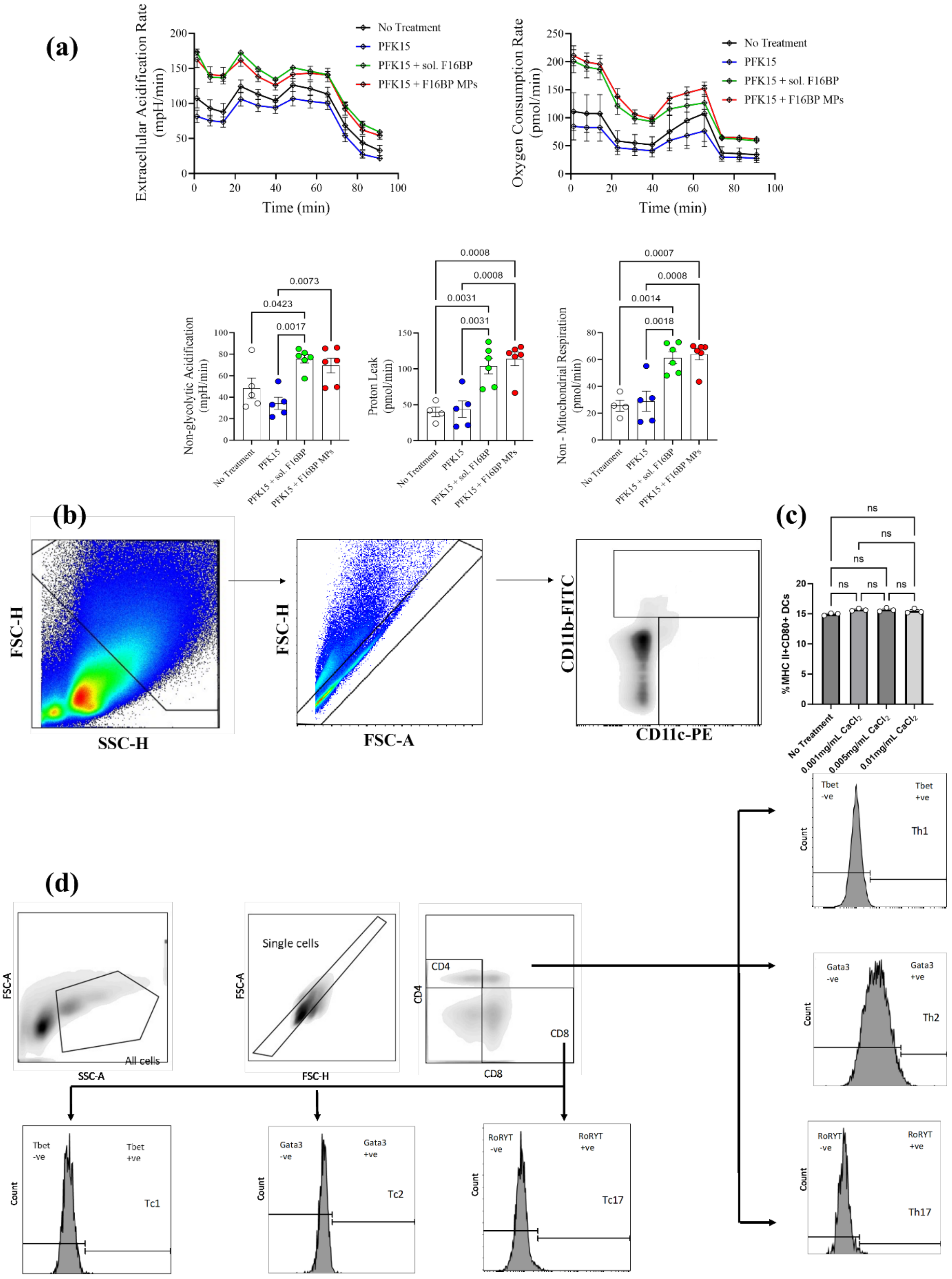

(a)
Extracellular Acidification Rate (mpH/min)
Time (min)
No Treatment
PFK15
PFK15 + sol. F16BP
PFK15 + F16BP MPs
Oxygen Consumption Rate (pmol/min)
Time (min)
No Treatment
PFK15
PFK15 + sol. F16BP
PFK15 + F16BP MPs
Non-glycolytic Acidification (mpH/min)
0.0073
0.0423
0.0017
Proton Leak (pmol/min)
0.0008
0.0008
0.0031
0.0031
Non - Mitochondrial Respiration (pmol/min)
0.0007
0.0008
0.0014
0.0018
No Treatment
PFK15
PFK15 + sol. F16BP
PFK15 + F16BP MPs
(b)
FSC-H
SSC-H
FSC-H
FSC-A
CD11b-FITC
CD11c-PE
(c)
ns
% MHC II+CD80+ DCs
No Treatment
0.001mg/mL CaCl2
0.005mg/mL CaCl2
0.01mg/mL CaCl2
(d)
FSC-A
SSC-A
All cells
Single cells
FSC-H
CD4
CD8
Tbet -ve
Tbet +ve
Th1
Gata3 -ve
Gata3 +ve
Th2
RoRYT -ve
RoRYT +ve
Th17
Tc1
Tc2
Tc17
Count

Figure S5: (a) Seahorse assay measurements for DCs treated with different treatment groups, (n=10; One-way ANOVA Tukey's test). Data represented as mean ± std err. **(b)** flow cytometry schema used for analysis of DCs and macrophages throughout the manuscript. **(c)** effect of Ca on DC activation. (n=5; One-way ANOVA Tukey's test). Data represented as mean ± std err. **(d)** flow cytometry schema used for analysis of T cells.

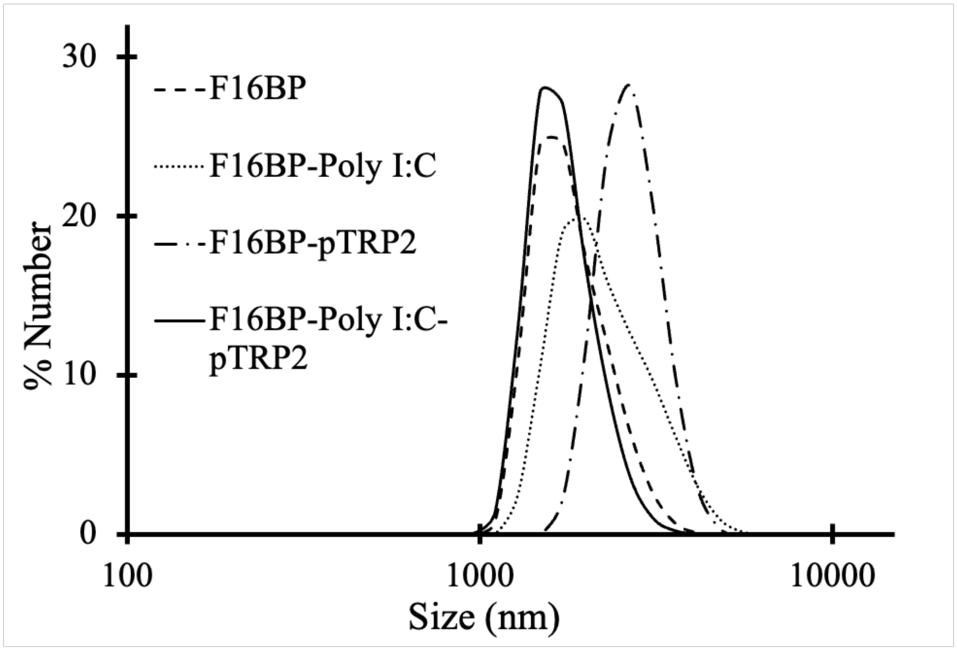

	F16BP	F16BP-PolyIC	F16BP-pTRP2	F16BP-PolyIC-pTRP2
Average (nm)	2343	2787	2984	2788
Std Dev (nm)	363	324	79	567

Figure S6: Microparticles were generated using F16BP, poly I:C and pTRP2.

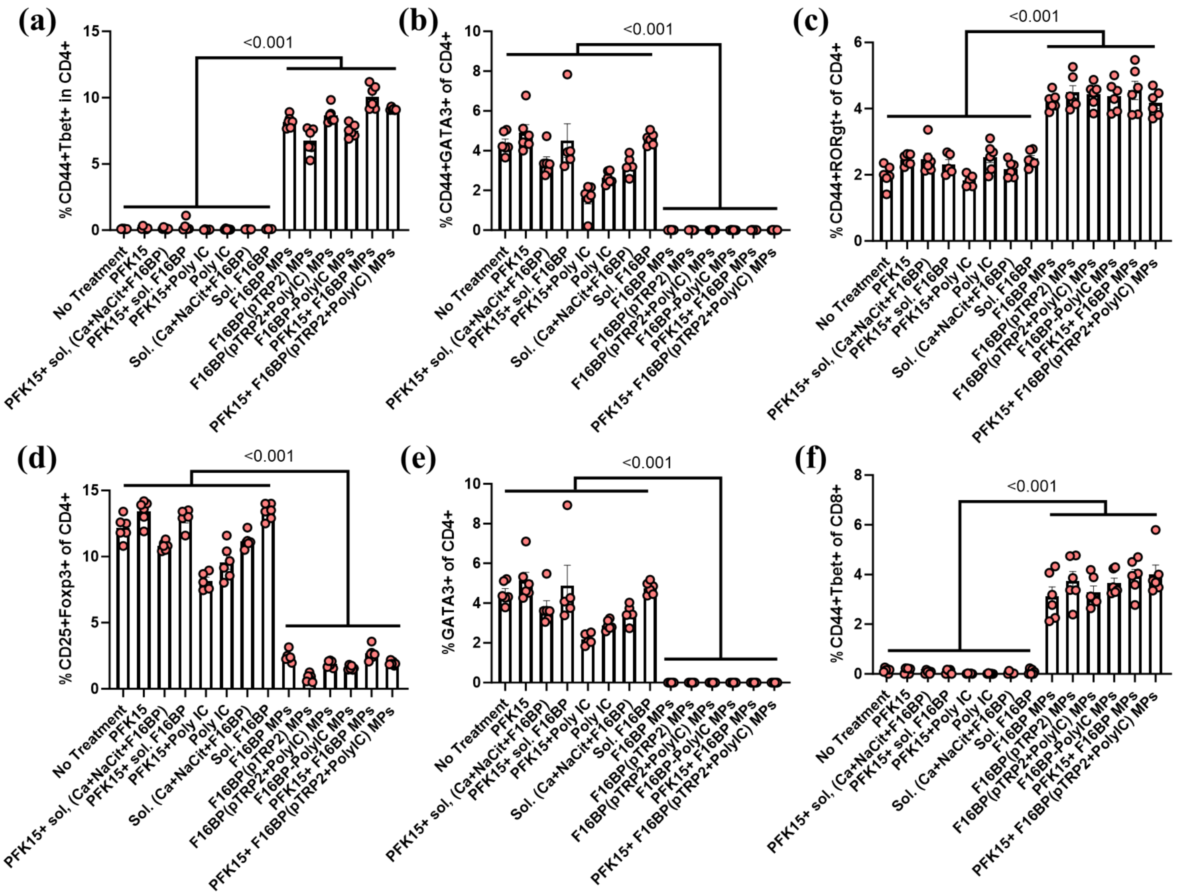

Figure S7: DCs treated with F16BP based formulations differentially lead to activation of pro-inflammatory T cells in a syngeneic mixed lymphocyte reaction. (a) Activated Th1; **(b)** Activated Th2; **(c)** Activation of Th17; **(d)** Regulatory T cells; **(e)** Th2; **(f)** Activated cytotoxic T cells (n=6; One-way ANOVA Tukey's test, * - p<0.05a). Data represented as mean ± std err.

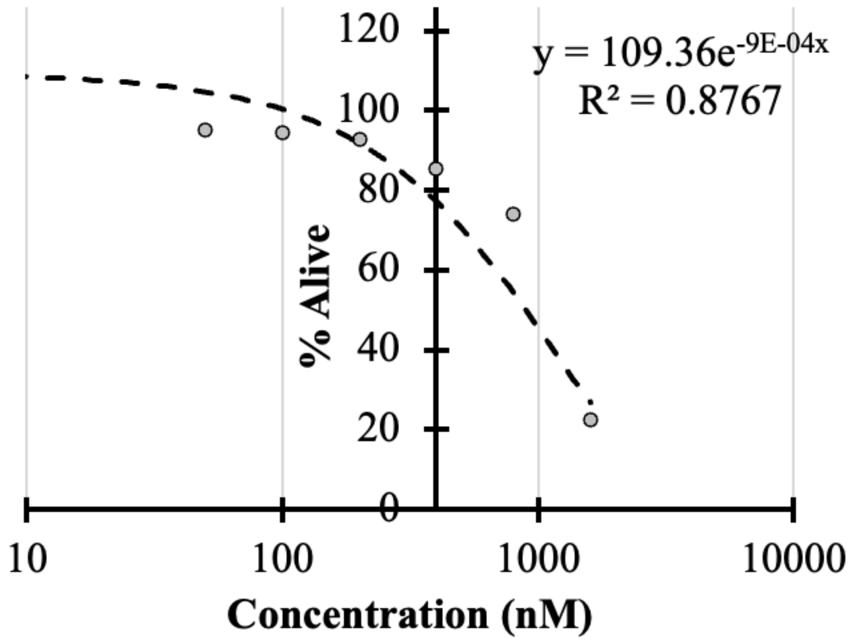

Figure S8: Proliferation assay using MTT, shows that PFK15 prevents growth of YUMM1.1 cancer cells (n=6). Data represented as mean ± std err.

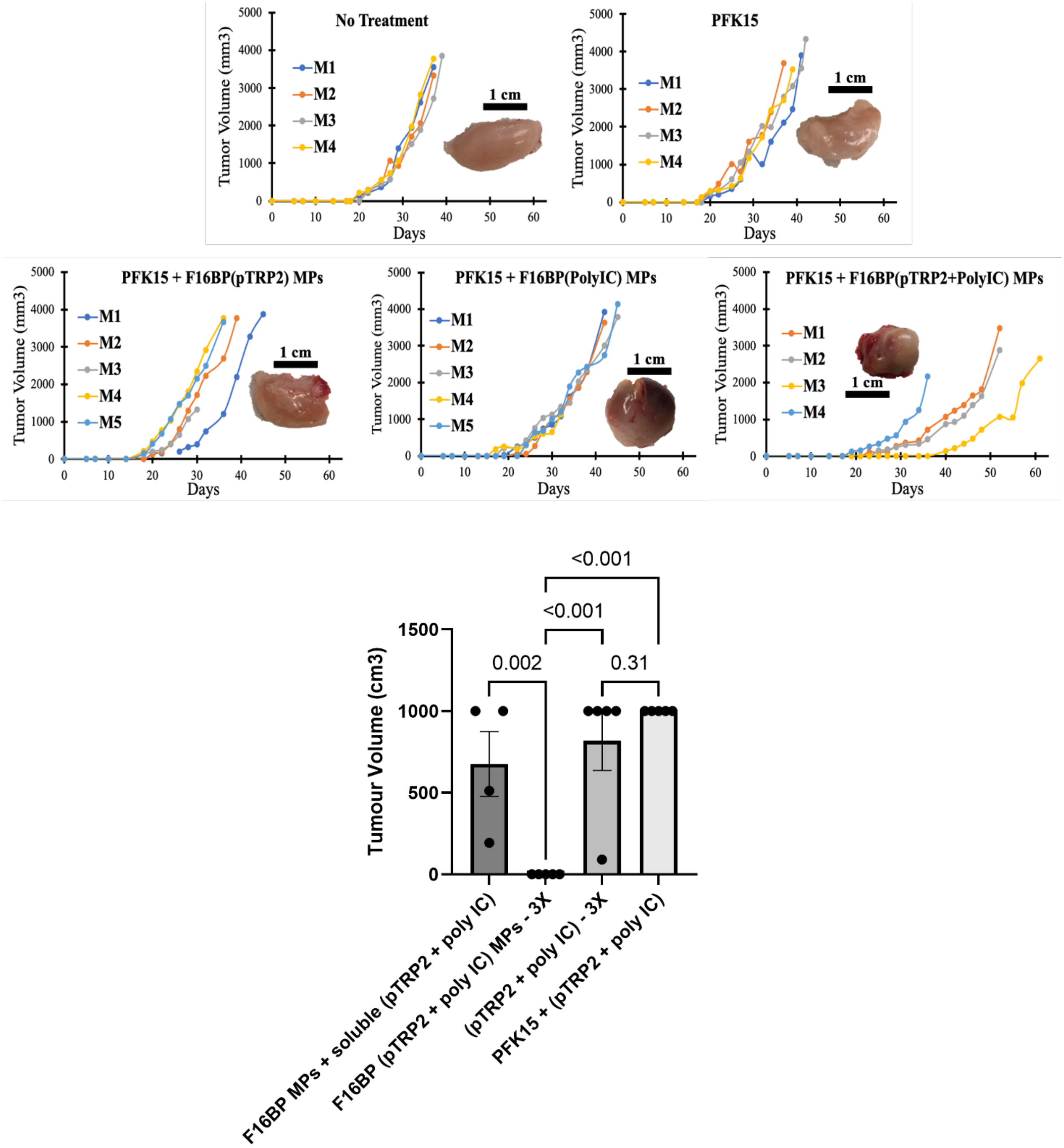

Figure S9: Individual tumour growth in mice treated with various treatment groups along with representative tumor images on day 35. **Bottom figure** – Three times (3X) the dose of F16BP(pTRP2+poly IC) prevented tumuor growth in mice till 60 days (tumour not detected)

whereas 3 times the dose of pTRP2 + poly IC, still led to generation of the tumour in mice. * - p value < 0.05 significantly different from all the groups; One way ANOVA, n = 5 per group.

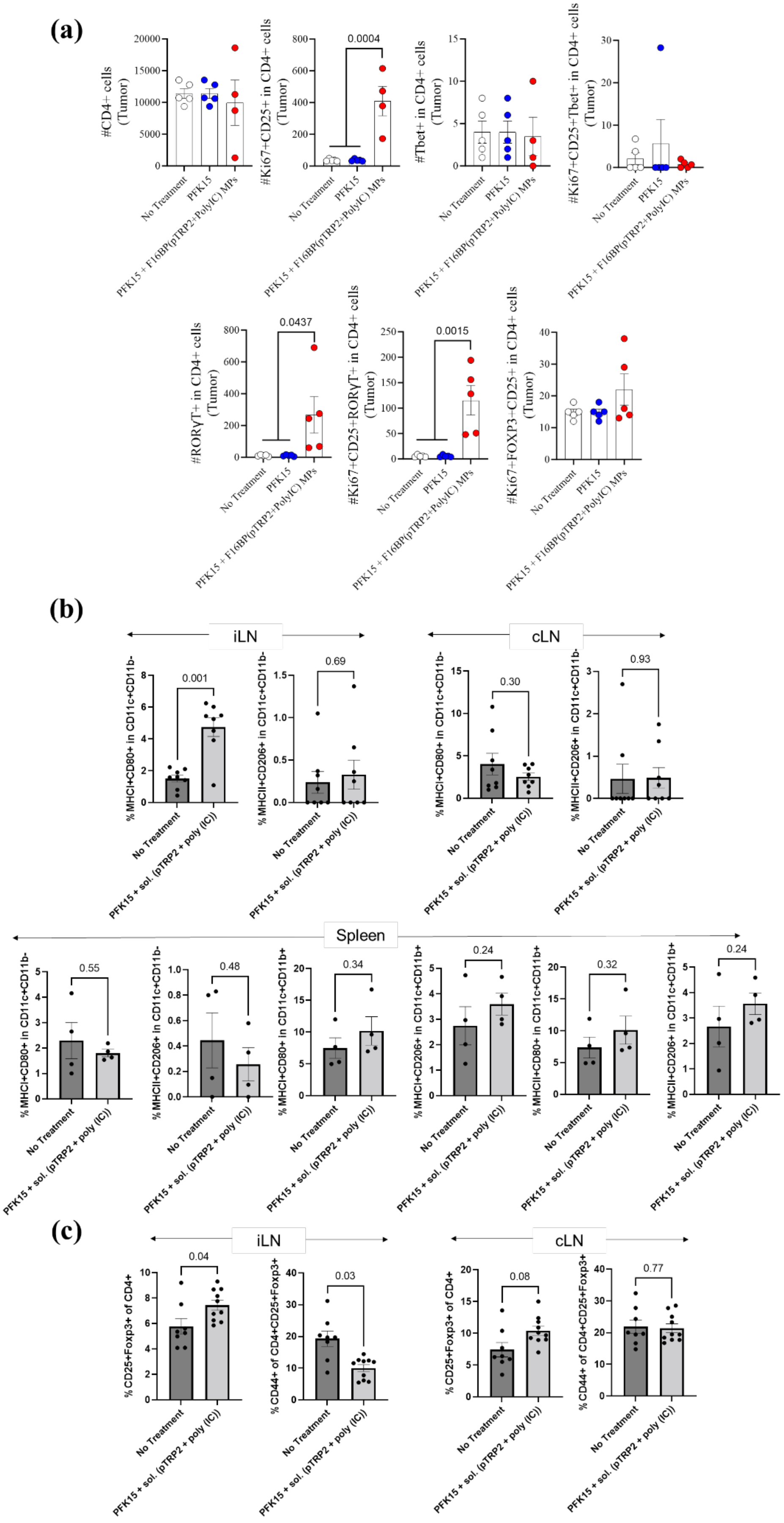

(a)
#CD4+ cells (Tumor)
#Ki67+CD25+ in CD4+ cells (Tumor)
0.0004
#Tbet+ in CD4+ cells (Tumor)
#Ki67+CD25+Tbet+ in CD4+ cells (Tumor)
No Treatment
PFK15
PFK15 + F16BP(pTRP2+PolyIC) MPs
#RORγT+ in CD4+ cells (Tumor)
0.0437
#Ki67+CD25+RORγT+ in CD4+ cells (Tumor)
0.0015
#Ki67+FOXP3−CD25+ in CD4+ cells (Tumor)
(b)
iLN
cLN
% MHCI+CD80+ in CD11c+CD11b-
0.001
% MHCII+CD206+ in CD11c+CD11b-
0.69
0.30
0.93
No Treatment
PFK15 + sol. (pTRP2 + poly (IC))
Spleen
0.55
0.48
% MHCI+CD80+ in CD11c+CD11b+
0.34
% MHCI+CD206+ in CD11c+CD11b+
0.24
% MHCII+CD80+ in CD11c+CD11b+
0.32
% MHCII+CD206+ in CD11c+CD11b+
0.24
(c)
iLN
cLN
% CD25+Foxp3+ of CD4+
0.04
% CD44+ of CD4+CD25+Foxp3+
0.03
0.08
0.77

Figure S10: (a) Subcutaneously injected Vacc MPs modulate adaptive immune responses in tumours, *in vivo* (n=5; One-way ANOVA Tukey's test). **(b)** In the inguinal lymph nodes (iLN) there was increased levels of activated DCs in the mice that were treated with PFK15 + soluble pTRP2 and soluble poly IC as compared to the no treatment group (n=4; One-way ANOVA Tukey's test). **(c)** In the inguinal lymph nodes (iLN) there was increased levels of Tregs, but decreased activated Tregs in the mice that were treated with PFK15 + soluble pTRP2 and soluble poly IC as compared to the no treatment group (n=10; One-way ANOVA Tukey's test).. Data represented as mean ± std err.

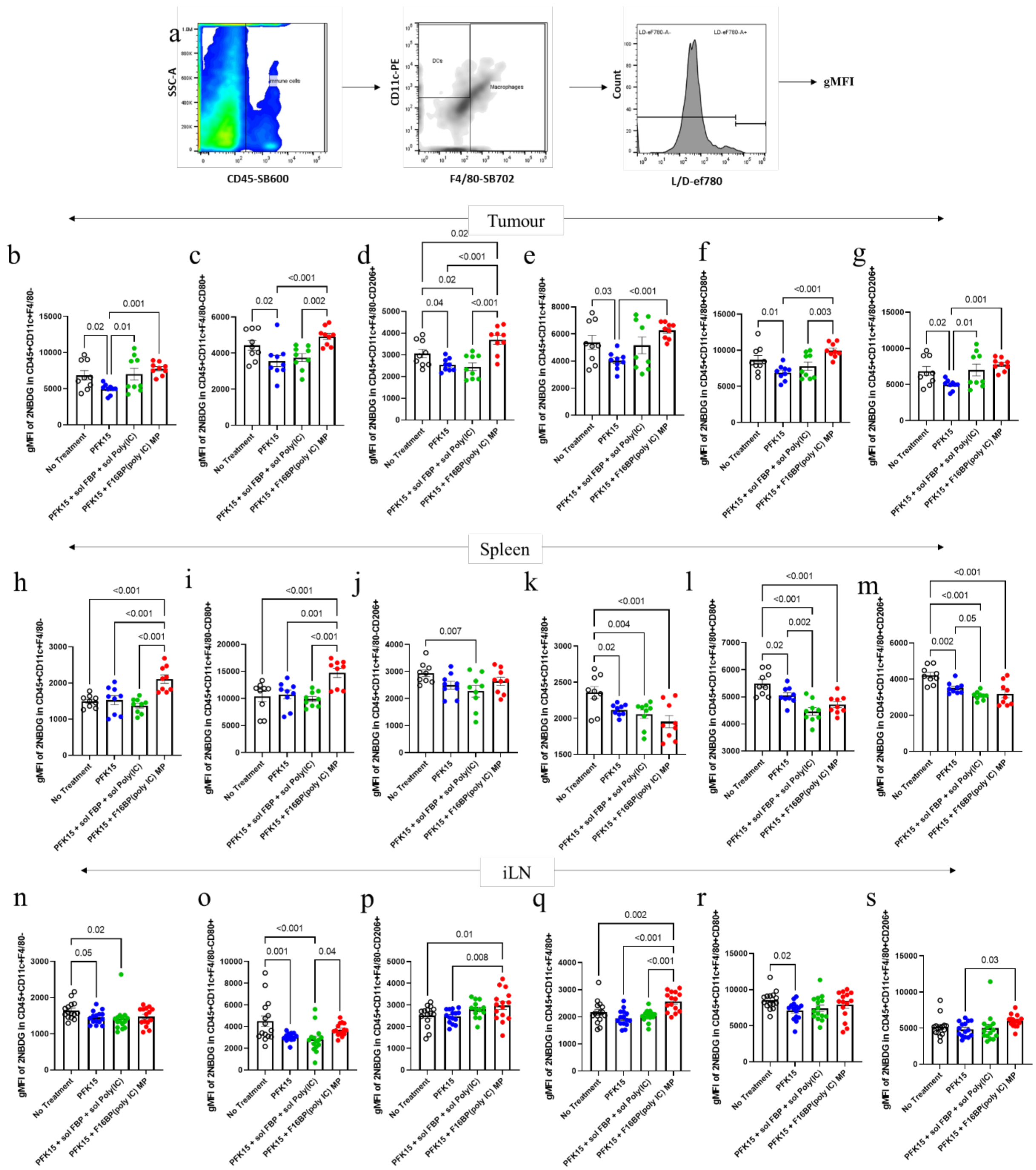

Figure S11: PFK15 inhibits innate immune cell glycolysis, whereas the F16BP-poly (IC) MPs are able to increase glycolysis in DCs and macrophages, and maintain this increase as measured using 2NBDG uptake assay on cells isolated from –

YUMM1.1 tumours - (**a**) Flow schematic; (**b**) dendritic cells; (**c**) activated dendritic cells; (**d**) immunosuppressive dendritic cells; (**e**) macrophages; (**f**) activated macrophages; (**g**) immunosuppressive macrophages. N = 9, avg±SEM, One-way ANOVA.

Spleen – (**h**) dendritic cells; (**i**) activated dendritic cells; (**j**) immunosuppressive dendritic cells; (**k**) macrophages; (**l**) activated macrophages; (**m**) immunosuppressive macrophages. N = 9, avg±SEM, One-way ANOVA.

Inguinal lymph nodes - (**n**) dendritic cells; (**o**) activated dendritic cells; (**p**) immunosuppressive dendritic cells; (**q**) macrophages; (**r**) activated macrophages; (**s**) immunosuppressive macrophages. N = 9, avg±SEM, One-way ANOVA.

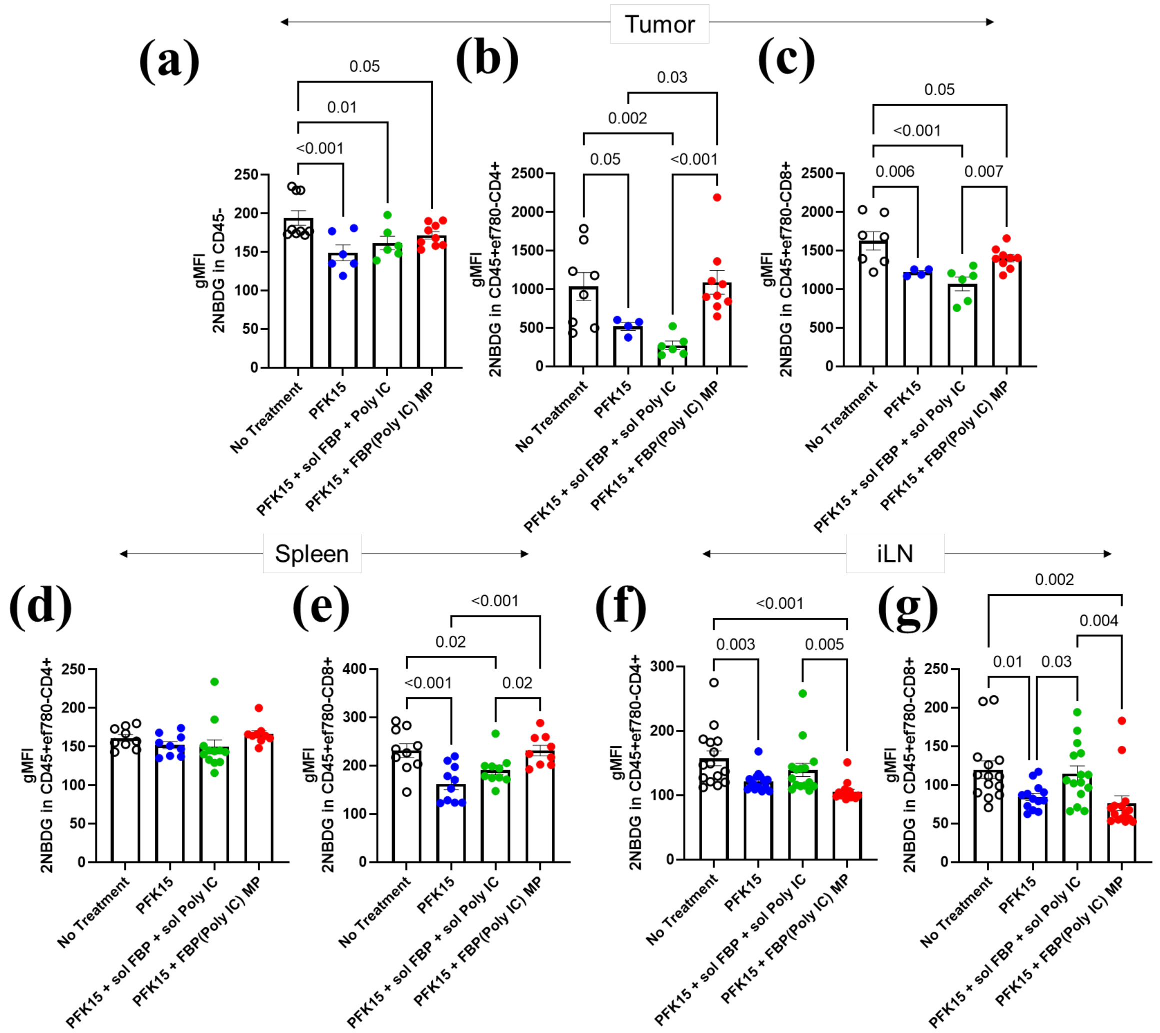

Figure S12: PFK15 inhibits adaptive T cell glycolysis, whereas the F16BP-poly (IC) MPs increase glycolysis, and maintain this increase as measured using 2NBDG uptake assay on cells isolated from

YUMM1.1 tumours - (**a**) cancer cells; (**b**) alive T helper cells; (**c**) alive cytotoxic T cells. N = 9, avg±SEM, One-way ANOVA.

Spleen – (**d**) alive T helper cells; (**e**) alive cytotoxic T cells. N = 9, avg±SEM, One-way ANOVA.

Inguinal lymph nodes - (**f**) alive T helper cells; (**g**) alive cytotoxic T cells. N = 9, avg±SEM, One-way ANOVA.

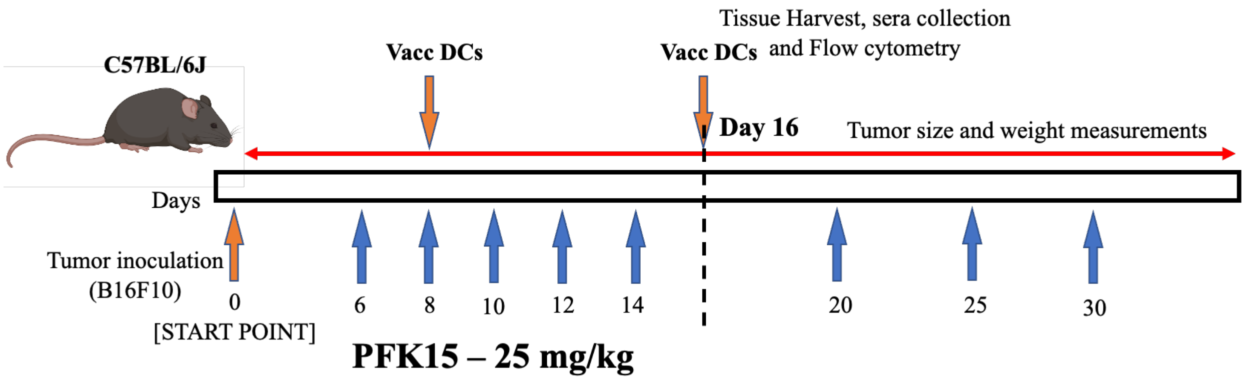

Figure S13: Study design for adoptive cell therapy model.

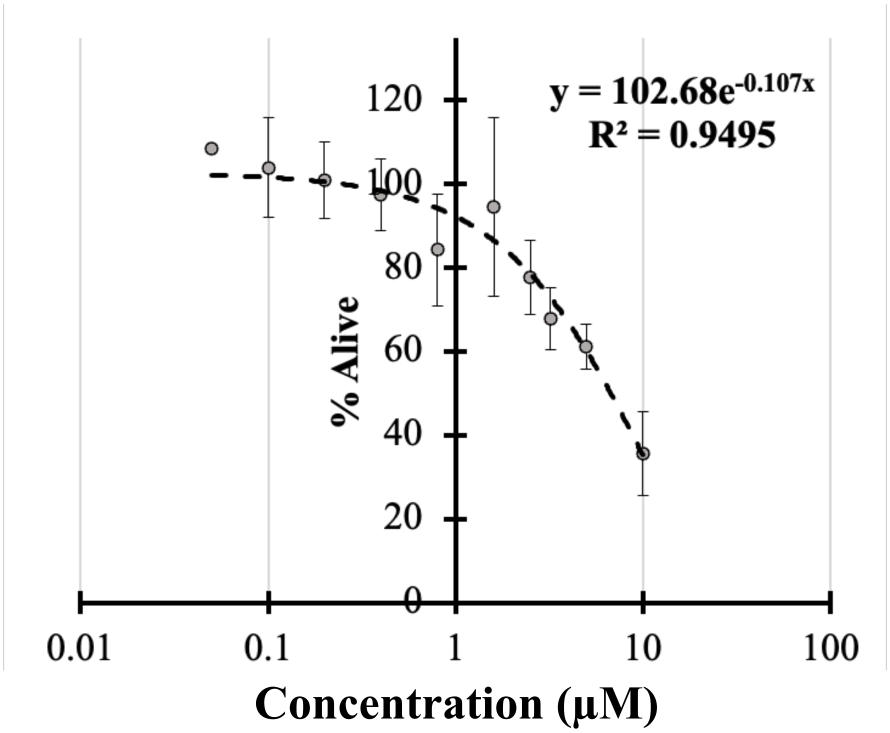

Figure S14: Proliferation assay using MTT, shows that PFK15 prevents growth of B16F10 cancer cells (n=6). Data represented as mean ± std err.

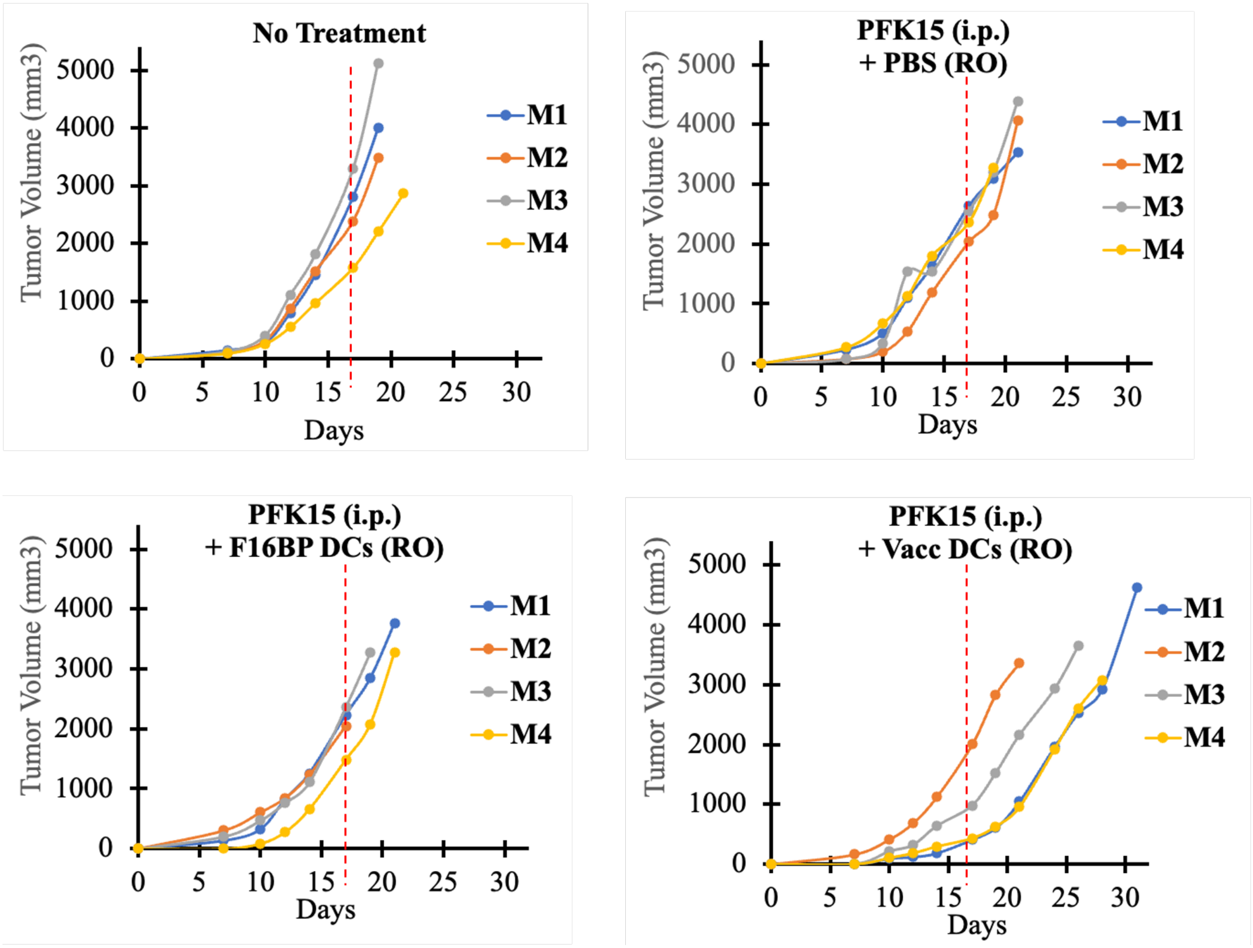

Figure S15: Individual tumor growth in mice treated with various treatment groups along with representative tumor images on day 35.

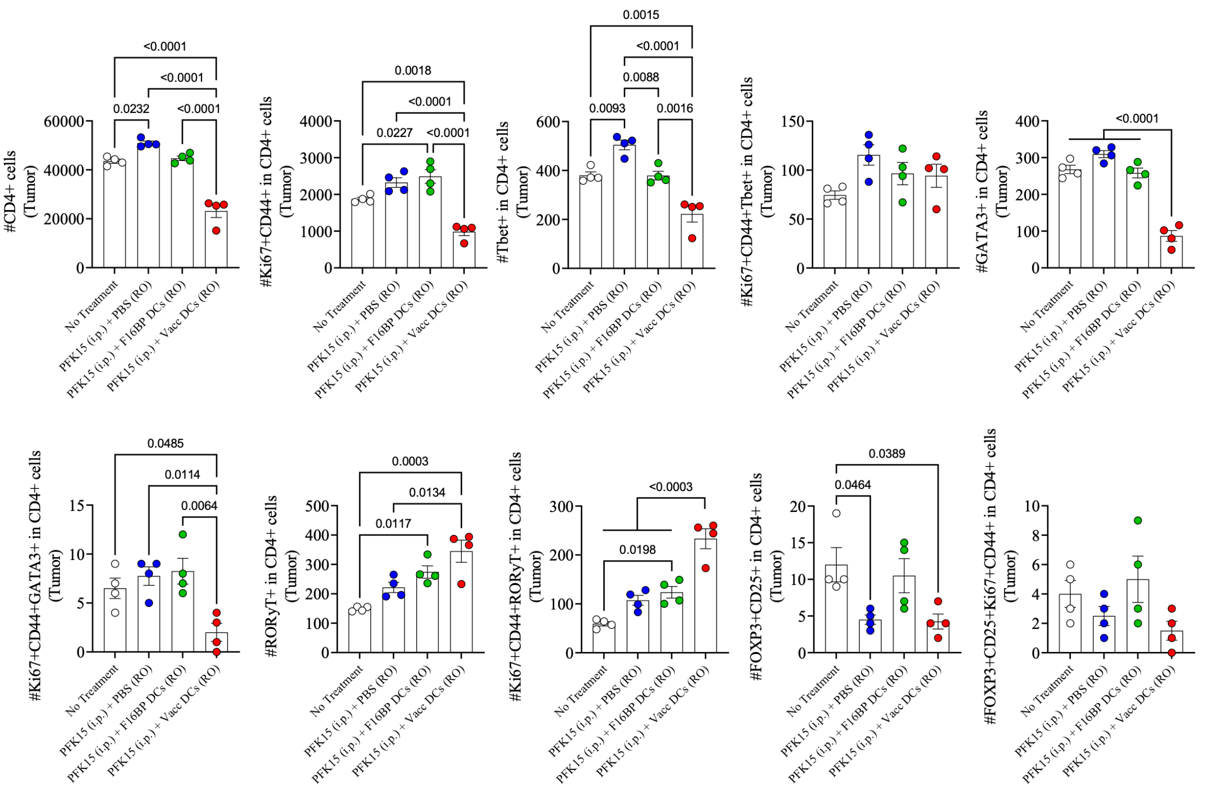

Figure S16: Adoptively transferred Vacc DCs modulate adaptive immune responses in tumours, *in vivo* (n=4; One-way ANOVA Tukey's test). Data represented as mean ± std err.

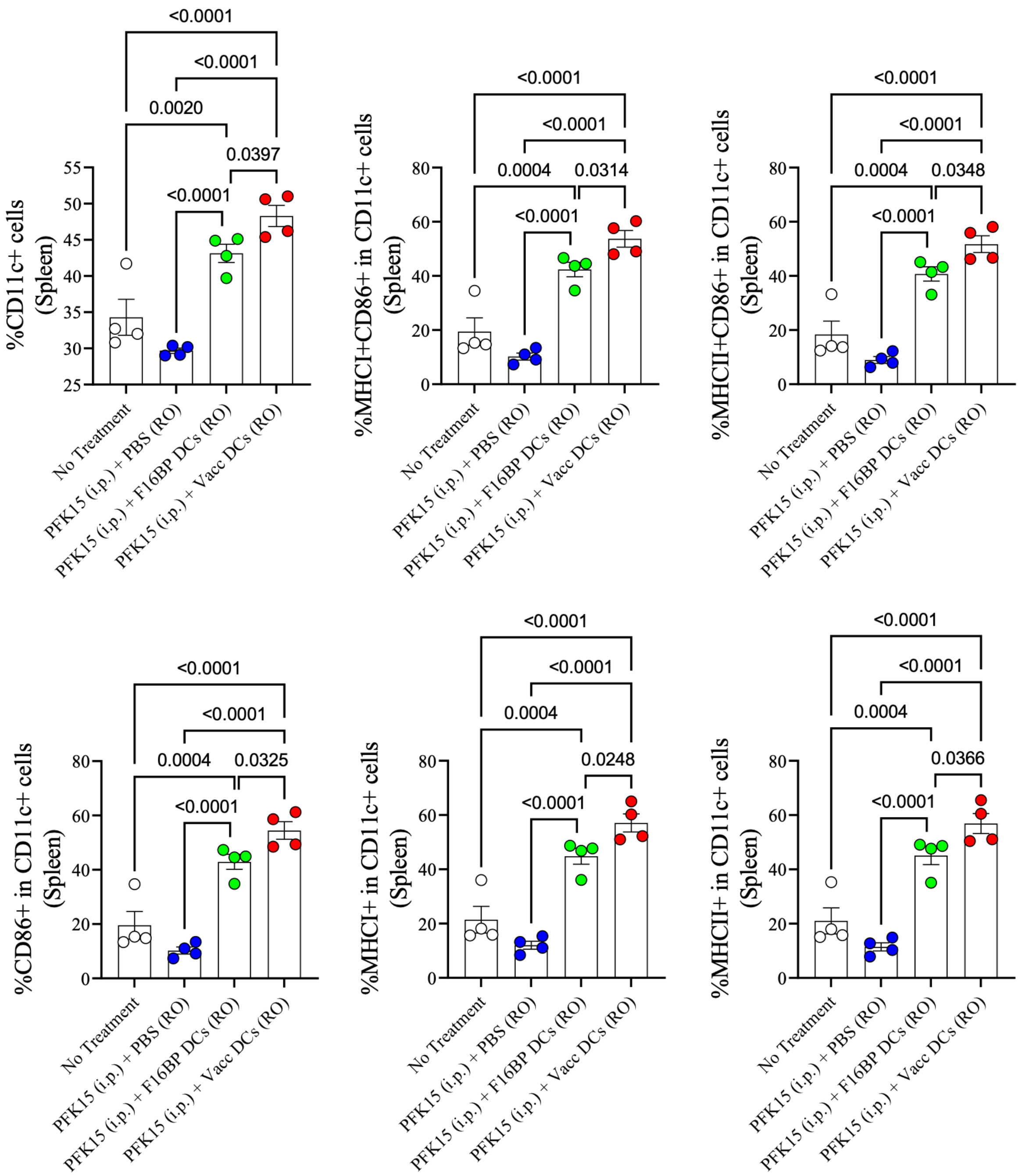

Figure S17: Adoptively transferred Vacc DCs modulate innate immune responses in spleen of mice treated with different groups, *in vivo* (n=4; One-way ANOVA Tukey's test). Data represented as mean ± std err.

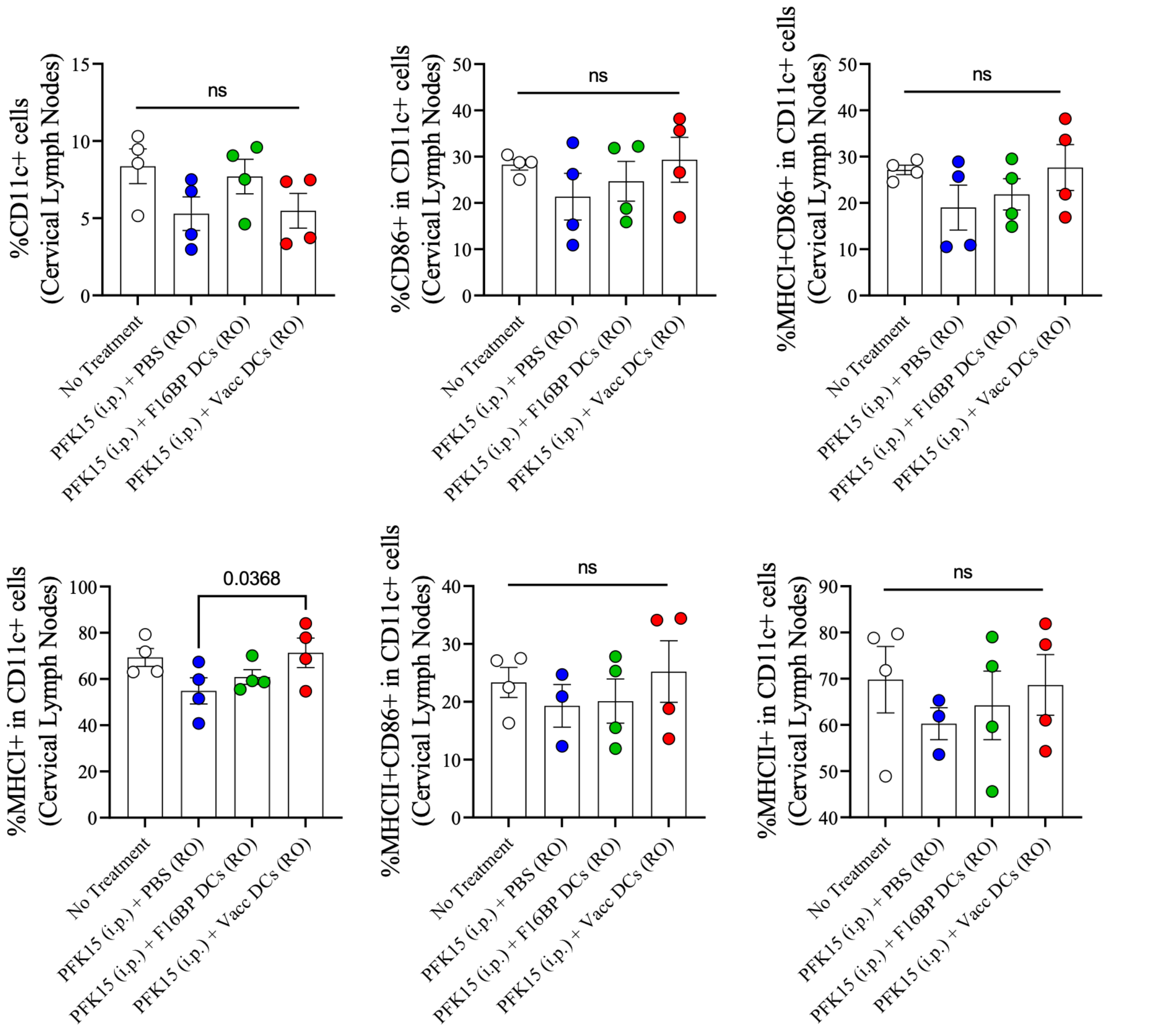

Figure S18: No significant differences were observed in innate immune responses in adoptively transferred Vacc DCs as compared to other treatment groups in the cervical lymph nodes, *in vivo* (n=4; One-way ANOVA Tukey's test). Data represented as mean ± std err.

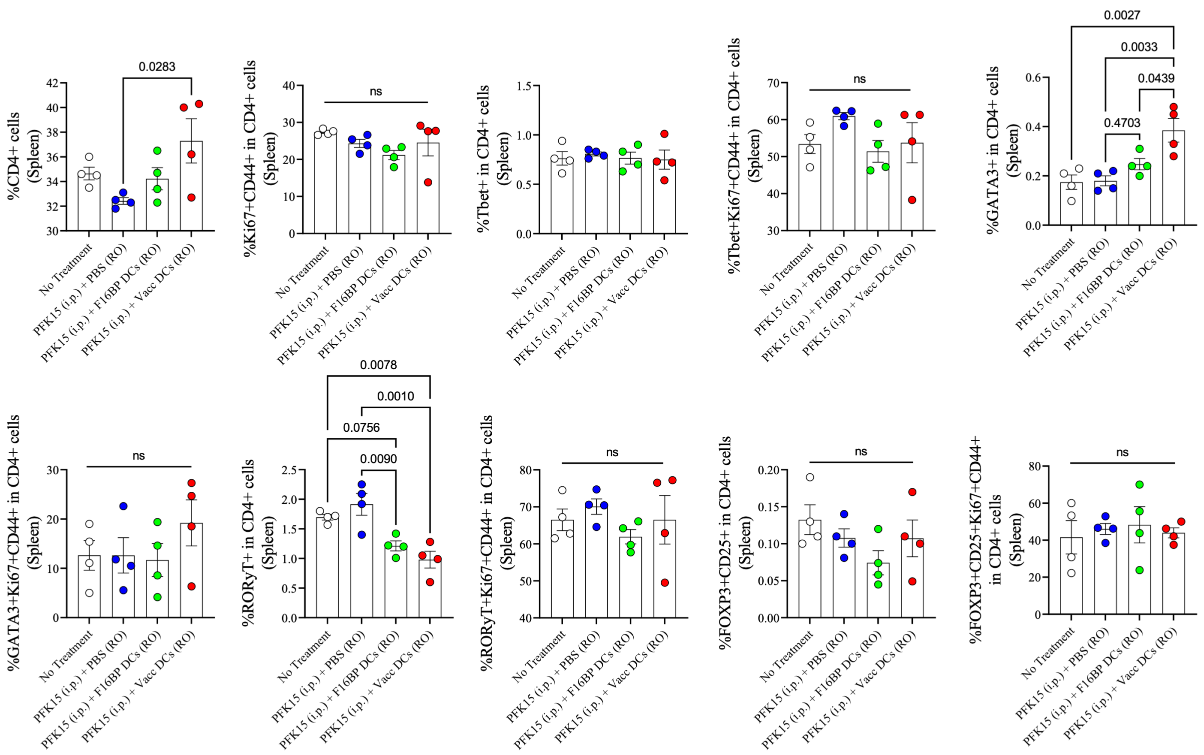

Figure S19: Adoptively transferred Vacc DCs modulate adaptive immune responses (helper T cell) in spleen of mice treated with different groups, *in vivo* (n=4; One-way ANOVA Tukey's test). Data represented as mean ± std err.

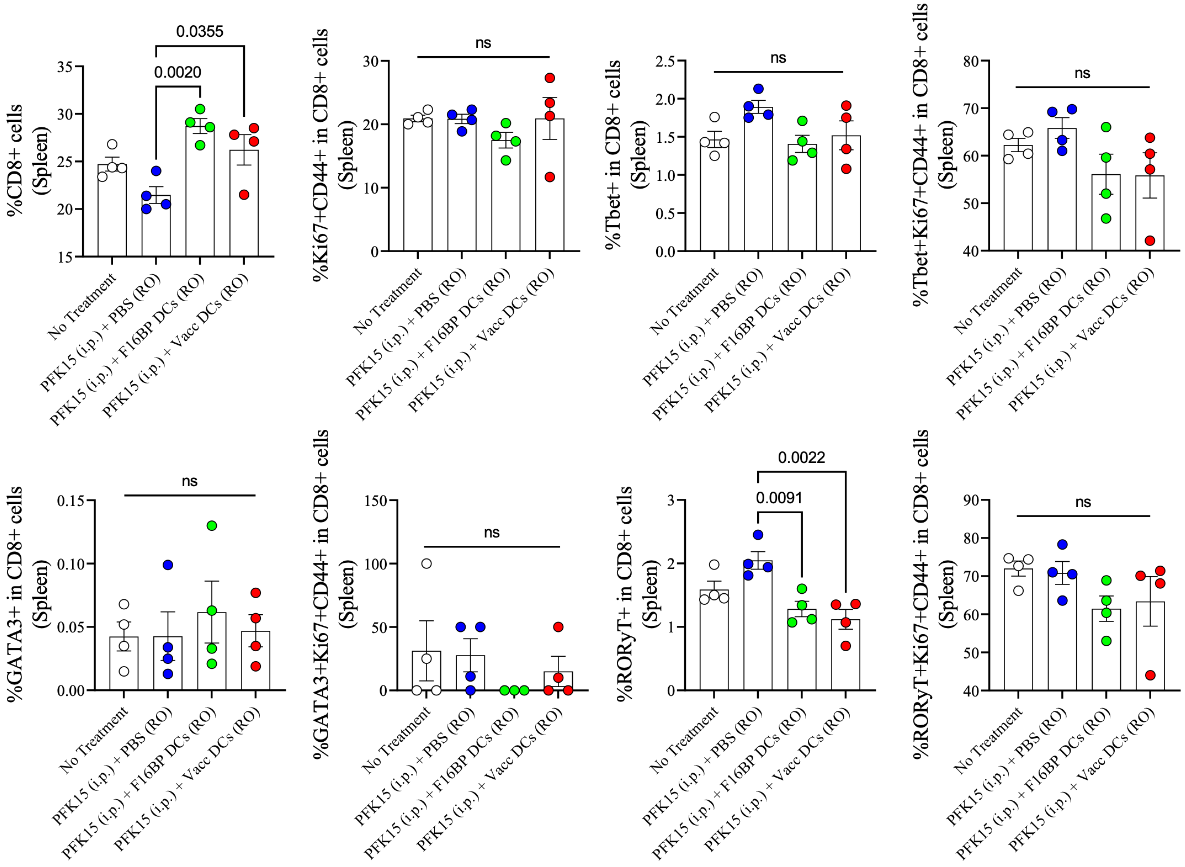

Figure S20: Adoptively transferred Vacc DCs modulate adaptive immune responses (cytotoxic T cell) in spleen of mice treated with different groups, *in vivo* (n=4; One-way ANOVA Tukey's test). Data represented as mean ± std err.

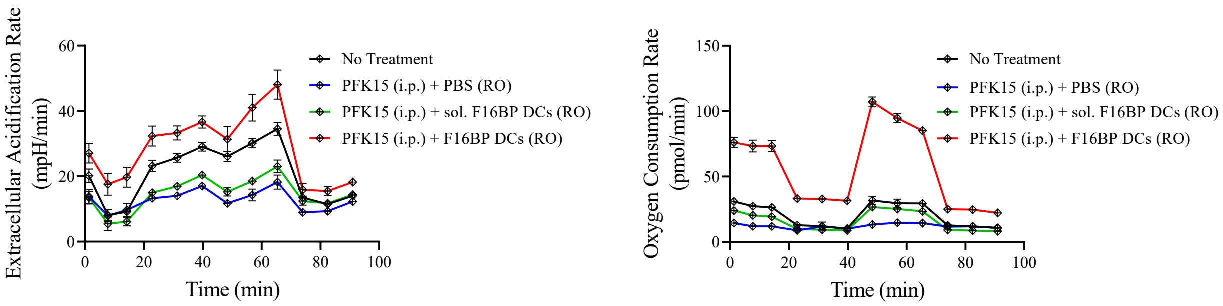

Figure S21: Seahorse assay measurements for DCs isolated from spleen of mice. (n=5; One-way ANOVA Tukey's test). Data represented as mean ± std err.

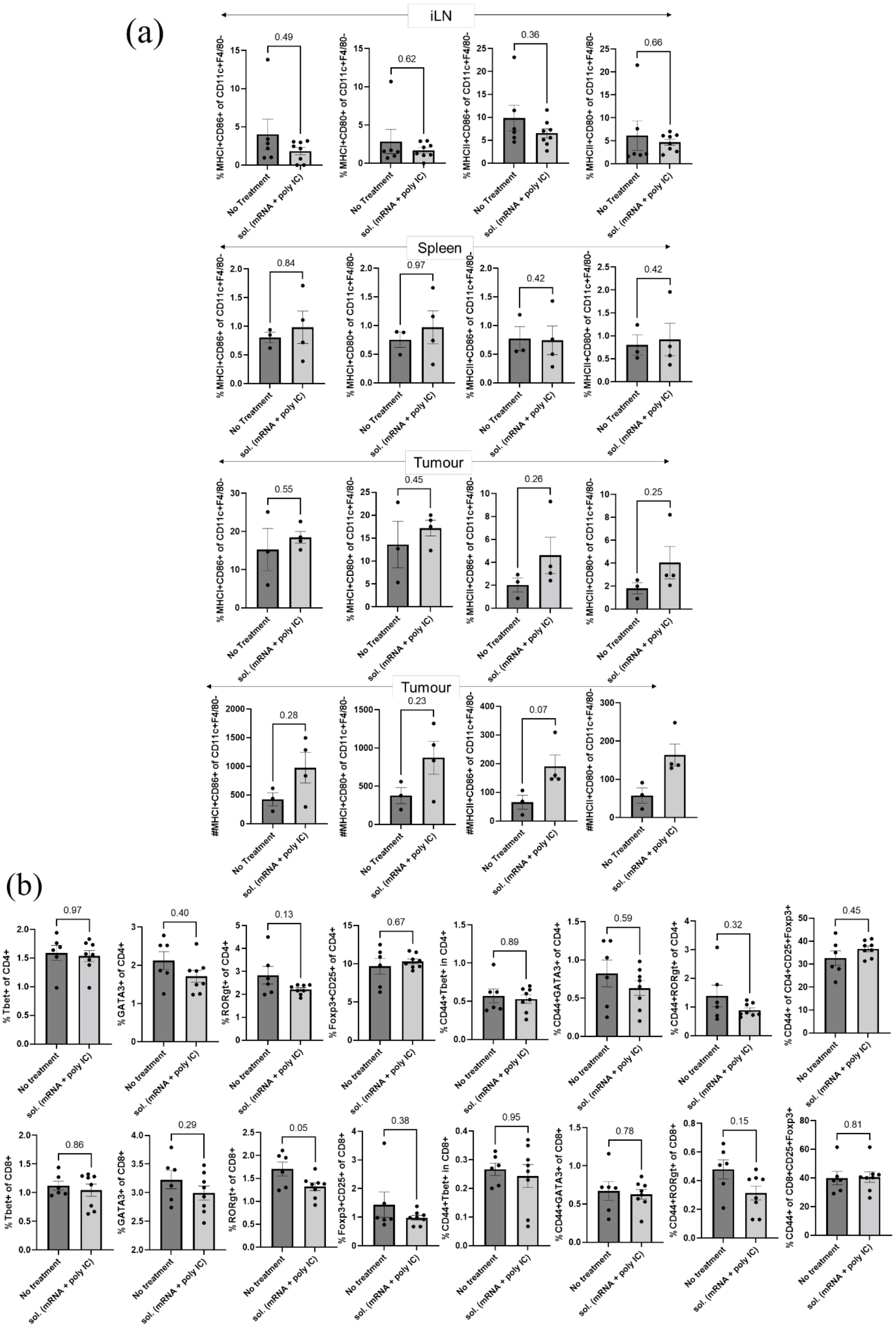
(a)
iLN
% MHCI+CD86+ of CD11c+F4/80-
0.49
% MHCI+CD80+ of CD11c+F4/80-
0.62
% MHCII+CD86+ of CD11c+F4/80-
0.36
% MHCII+CD80+ of CD11c+F4/80-
0.66
Spleen
% MHCI+CD86+ of CD11c+F4/80-
0.84
% MHCI+CD80+ of CD11c+F4/80-
0.97
% MHCII+CD86+ of CD11c+F4/80-
0.42
% MHCII+CD80+ of CD11c+F4/80-
0.42
Tumour
% MHCI+CD86+ of CD11c+F4/80-
0.55
% MHCI+CD80+ of CD11c+F4/80-
0.45
% MHCII+CD86+ of CD11c+F4/80-
0.26
% MHCII+CD80+ of CD11c+F4/80-
0.25
Tumour
#MHCI+CD86+ of CD11c+F4/80-
0.28
#MHCI+CD80+ of CD11c+F4/80-
0.23
#MHCII+CD86+ of CD11c+F4/80-
0.07
#MHCII+CD80+ of CD11c+F4/80-
No Treatment
sol. (mRNA + poly IC)
(b)
% Tbet+ of CD4+
0.97
% GATA3+ of CD4+
0.40
% RORgt+ of CD4+
0.13
% Foxp3+CD25+ of CD4+
0.67
% CD44+Tbet+ in CD4+
0.89
% CD44+GATA3+ of CD4+
0.59
% CD44+RORgt+ of CD4+
0.32
% CD44+ of CD4+CD25+Foxp3+
0.45
% Tbet+ of CD8+
0.86
% GATA3+ of CD8+
0.29
% RORgt+ of CD8+
0.05
% Foxp3+CD25+ of CD8+
0.38
% CD44+Tbet+ in CD8+
0.95
% CD44+GATA3+ of CD8+
0.78
% CD44+RORgt+ of CD8+
0.15
% CD44+ of CD8+CD25+Foxp3+
0.81
No treatment
sol. (mRNA + poly IC)

Figure S22: No significant differences found for (a) DCs (b) T cells when mice were treated with DCs loaded with soluble mRNA and soluble poly (IC) as compared to no treatment. (n = 4 for DCs; n=6 for T cells; One-way ANOVA Tukey's test). Data represented as mean ± std err.
