# Supplementary material for: Rescue of dendritic cells from glycolysis inhibition improves cancer immunotherapy in mice
Source: Nat Commun. 2023 Sep 2;14:5333. doi: 10.1038/s41467-023-41016-z (PMC10475105; doi:10.1038/s41467-023-41016-z)
Supplement: Supplementary file 3 — Reporting Summary [file 41467_2023_41016_MOESM3_ESM.pdf]

nature portfolio

Corresponding author(s): Abhinav P. Acharya

Last updated by author(s): Jul 31, 2023

Reporting Summary

Nature Portfolio wishes to improve the reproducibility of the work that we publish. This form provides structure for consistency and transparency in reporting. For further information on Nature Portfolio policies, see our Editorial Policies and the Editorial Policy Checklist.

Statistics

For all statistical analyses, confirm that the following items are present in the figure legend, table legend, main text, or Methods section.

n/a	Confirmed	
☐	☒	The exact sample size (*n*) for each experimental group/condition, given as a discrete number and unit of measurement
☐	☒	A statement on whether measurements were taken from distinct samples or whether the same sample was measured repeatedly
☐	☒	The statistical test(s) used AND whether they are one- or two-sided<br>*Only common tests should be described solely by name; describe more complex techniques in the Methods section.*
☐	☒	A description of all covariates tested
☐	☒	A description of any assumptions or corrections, such as tests of normality and adjustment for multiple comparisons
☐	☒	A full description of the statistical parameters including central tendency (e.g. means) or other basic estimates (e.g. regression coefficient) AND variation (e.g. standard deviation) or associated estimates of uncertainty (e.g. confidence intervals)
☐	☒	For null hypothesis testing, the test statistic (e.g. *F*, *t*, *r*) with confidence intervals, effect sizes, degrees of freedom and *P* value noted<br>*Give P values as exact values whenever suitable.*
☒	☐	For Bayesian analysis, information on the choice of priors and Markov chain Monte Carlo settings
☒	☐	For hierarchical and complex designs, identification of the appropriate level for tests and full reporting of outcomes
☒	☐	Estimates of effect sizes (e.g. Cohen's *d*, Pearson's *r*), indicating how they were calculated

Our web collection on statistics for biologists contains articles on many of the points above.

Software and code

Policy information about availability of computer code

Data collection	No software was used for data collection.
Data analysis	Flowjo v10, GraphPad Prism software 9.0

For manuscripts utilizing custom algorithms or software that are central to the research but not yet described in published literature, software must be made available to editors and reviewers. We strongly encourage code deposition in a community repository (e.g. GitHub). See the Nature Portfolio guidelines for submitting code & software for further information.

Data

Policy information about availability of data

All manuscripts must include a data availability statement. This statement should provide the following information, where applicable:

- Accession codes, unique identifiers, or web links for publicly available datasets
- A description of any restrictions on data availability
- For clinical datasets or third party data, please ensure that the statement adheres to our policy

All the raw data is available through source data file, any further data needed can be obtained upon request from the corresponding author.

Human research participants

Policy information about studies involving human research participants and Sex and Gender in Research.

Reporting on sex and gender	N/A
Population characteristics	N/A
Recruitment	N/A
Ethics oversight	N/A

Note that full information on the approval of the study protocol must also be provided in the manuscript.

Field-specific reporting

Please select the one below that is the best fit for your research. If you are not sure, read the appropriate sections before making your selection.

☒ Life sciences ☐ Behavioural & social sciences ☐ Ecological, evolutionary & environmental sciences

For a reference copy of the document with all sections, see nature.com/documents/nr-reporting-summary-flat.pdf

Life sciences study design

All studies must disclose on these points even when the disclosure is negative.

Sample size	A minimum of 4 for in vivo and minimum of 5 for in vitro independent replicates were used for all samples unless otherwise specified in the manuscript. This sample size was determined to be sufficient for calculating average and standard error and eventually statistical significance amongst different groups.<br>Sample sizes were chosen by performing power analysis with an alpha of 0.05 and a power of 80%.
Data exclusions	A pre-established criteria was set for all the data points in this study. Specifically, significant outliers, as determined using graphpad (alpha = 0.05) from a single group were excluded.
Replication	All in vitro experiments were replicated independently at least 5 times, whereas, all in vivo experiments were independently replicated at least 4 times unless otherwise specified in the manuscript.
Randomization	In vitro treatment of cells was done randomly (5 or more independent replicates unless otherwise specified in the manuscript). For in vivo studies, mice were housed in random cages before experiments.
Blinding	For in vivo studies, tumour inoculation, treatments, tumour measurement and analysis was conducted by different investigators and the investigator providing the treatment was not blinded since this is not possible, however, in each of the subsequent steps the scientists were not aware of the treatment groups. Adoptive transfer study was performed in a blinded fashion.

Reporting for specific materials, systems and methods

We require information from authors about some types of materials, experimental systems and methods used in many studies. Here, indicate whether each material, system or method listed is relevant to your study. If you are not sure if a list item applies to your research, read the appropriate section before selecting a response.

Materials & experimental systems

n/a	Involved in the study	
☐	☒	Antibodies
☐	☒	Eukaryotic cell lines
☒	☐	Palaeontology and archaeology
☐	☒	Animals and other organisms
☒	☐	Clinical data
☒	☐	Dual use research of concern

Methods

n/a	Involved in the study	
☒	☐	ChIP-seq
☐	☒	Flow cytometry
☒	☐	MRI-based neuroimaging

Antibodies

Antibodies used

Target Fluorophore Company Catalog # Clone
CD4 PE BD 12-0041-82 GK1.5
CD8 APC-R700 BD 564983 53-6.7
CD25 PECy7 BD 552880 PC61
CD11c PE BioLegend 117308 N418
CD86 SB600 Thermo 63-0862-82 GL1
CD80 PE-Cy5 Invitrogen 15-0801-82 16-10A1
MHCII APC BioLegend 107614 M5/114.15.2
Tbet BV785 BioLegend 644835 4B10
FoxP3 eF450 Invitrogen 48-5773-82 FJK-16s
RORgT BV650 BD 564722 Q31-378
Ki67 FITC Invitrogen 11-5698-82 SolA15
GATA3 BV711 BD 565449 L50-823
CD16/CD32:Fc Block NA Tonbo 70-0161-M001 2.4G2
F4/80 BV702 Invitrogen 67-4801-80 BM8
IL12 V450 BD 561456 C15.6
IFNY PE Tonbo 50-7311-U100 XMG1.2
IL10 PE/DAZZLE BioLegend 505034 JES5-16E3
TNFa BV510 BD 563386 MP6-XT22
CD11b FITC Tonbo 35-0112-U500 M1/70
CD3 NA BioLegend 100202 17A2
CD28 NA Tonbo 70-0281-U100 37.51
CD163 SB436 ThermoFisher 62-1631-82 TNKUPJ
CD206 PECy7 ThermoFisher 25-2061-82 MR6F3
CD3 Biolegend 100202 17A2
CD28 Tonbo Bioscences 70-0281-U100 37.51

Validation

Relative expression
This Antibody was verified by Relative expression to ensure that the antibody binds to the antigen stated.
CD4 Antibody
Staining of mouse splenocytes. As expected based on known relative expression patterns, CD4 clone GK1.5 stains about 25% of total lymphocytes and does not stain CD45R (B220)+ B cells. Details: Balb/c splenocytes were surface stained with CD45R (B220, clone RA3-6B2) and Rat IgG2b Isotype Control or CD4 (clone GK1.5). Cells in the lymphocyte gate were used for analysis.

CD8 Antibody
This antibody was conjugated to BD Horizon APC-R700, which has been developed exclusively by BD Biosciences as a better alternative to Alexa Fluor® 700. APC-R700 excites and emits at similar wavelengths to Alexa Fluor® 700 yet exhibits significantly improved brightness. This dye can be excited by the red laser and detected with the same filter set as Alexa Fluor® (eg, 730/45-nm filter).

CD25 Antibody
PE-Cy7 dye is a part of the BD PE family of dyes. This tandem fluorochrome is co
mprised of a R-Phycoerythrin (PE) donor that has excitation maxima (Ex Max) of 496-nm and 566-nm and an acceptor dye, Cy™7, with an emission maximum (Em Max) at 781-nm. PE can be excited by the Blue (488-nm), Green (532-nm) and yellow-green (561-nm) lasers and detected using an optical filter centered near 781 nm (e.g., a 760/60-nm bandpass filter). The donor dye can be excited by the Blue (488-nm), Green (532-nm) and yellow-green (561-nm) lasers and the acceptor dye can be excited by the Red (627–640-nm) laser resulting in cross-laser excitation and fluorescence spillover. Please ensure that your instrument's configurations (lasers and optical filters) are appropriate for this dye.

CD11c
Each lot of this antibody is quality control tested by immunofluorescent staining with flow cytometric analysis. For flow cytometric staining, the suggested use of this reagent is ≤ 0.25 µg per 106 cells in 100 µl volume. It is recommended that the reagent be titrated for optimal performance for other applications.

CD86
CD86 (B7-2) Antibody (63-0862-82) in Flow Staining of 3-day LPS (Product # 00-4976-03)-stimulated mouse splenocytes with Anti-Mouse CD19 FITC (Product # 11-0193-82) and 0.06 µg of Rat IgG2a K Isotype Control Super Bright 600 (Product # 63-4321-82) (left) or 0.06 µg of Anti-Mouse CD86 (B7-2) Super Bright 600 (right). Viable cells in the lymphocyte gate, as determined by Fixable Viability Dye eFluor® 780 (Product # 65-0865-14), were used for analysis.

CD80
Staining of 3-day LPS activated C57Bl/6 splenocytes with 0.03 µg of Armenian Hamster IgG Isotype Control PE-Cyanine5 (Product # 15-4888-82) (blue histogram) or 0.03 µg of Anti-Mouse CD80 (B7-1) PE-Cyanine5 (purple histogram).Total viable cells were used for analysis.

MHCII
Each lot of this antibody is quality control tested by immunofluorescent staining with flow cytometric analysis. For immunofluorescent staining, the suggested use of this reagent is ≤ 0.25 µg per 106cells in 100 µl volume. It is recommended that the reagent be titrated for optimal performance for each application.

Tbet
Each lot of this antibody is quality control tested by intracellular immunofluorescent staining using our True-Nuclear™ Transcription Factor Staining Protocol. Each lot of this antibody is quality control tested by immunofluorescent staining with flow cytometric analysis. For flow cytometric staining, the suggested use of this reagent is ≤0.5 µg per million cells in 100 µl volume. It is recommended that the reagent be titrated for optimal performance for each application.

FOXP3
Intracellular staining of mouse splenocytes. As expected based on known relative expression patterns, Foxp3 clone FJK-16s stains a subset of the CD4+ T cells and does not stain the CD8+ T cells. Details: Balb/c splenocytes were surface stained with CD3 (clone 17A2), CD4 (clone GK1.5) and CD8 (clone 53-6.7), followed by intracellular staining with Foxp3 (clone FJK-16s) using the Foxp3/ Transcription Factor Staining Buffer Set and protocol. Lymphocytes in the CD3+CD8+ (blue histogram) and CD3+CD4+ (purple histogram) gates were used for analysis.

RORgT
The BD Horizon Brilliant Violet™ 650 (BV650) Dye is part of the BD Horizon Brilliant Violet™ family of dyes. This tandem fluorochrome is comprised of a BV421 donor with an excitation maximum (Ex Max) of 406-nm and an acceptor dye with an emission maximum (Em Max) at 649-nm. BV650, driven by BD innovation, is designed to be excited by the violet laser (405-nm) and detected using an optical filter centered near 650-nm (e.g., a 660/20-nm bandpass filter). The acceptor dye can be excited by the Red (628–640-nm) laser resulting in cross-laser excitation and fluorescence spillover. Please ensure that your instrument's configurations (lasers and optical filters) are appropriate for this dye.

Ki67
Description: The monoclonal antibody SolA15 recognizes mouse and rat Ki-67, a 300 kDa nuclear protein. Ki-67 is present during all active phases of the cell cycle (G1, S, G2, and mitosis), but is absent from resting cells (G0). Ki-67 is detected within the nucleus during interphase but redistributes to the chromosomes during mitosis. Ki-67 is used as a marker for determining the growth fraction of a given population of cells. In studies of tumor cells, the "Ki-67 labeling index" refers to the number of Ki-67 positive cells within the population and this is used to predict outcome of particular cancer types. Ki-67 has been shown to interact with the DNA-bound protein chromobox protein homolog 3 (CBX3) (heterochromatin).

GATA3
The BD Horizon Brilliant Violet™ 711 (BV711) Dye is part of the BD Horizon Brilliant Violet™ family of dyes. This tandem fluorochrome is comprised of a BV421 donor with an excitation maximum (Ex Max) of 407-nm and an acceptor dye with an emission maximum (Em Max) at 713-nm. BV711, driven by BD innovation, is designed to be excited by the violet laser (405-nm) and detected using an optical filter centered near 710-nm (e.g., a 712/20-nm bandpass filter). The acceptor dye can be excited by the Red (628–640-nm) laser resulting in cross-laser excitation and fluorescence spillover. Please ensure that your instrument's configurations (lasers and optical filters) are appropriate for this dye.

F4/80
Staining of mouse resident peritoneal exudate cells with CD11b Monoclonal Antibody, PE (Product # 12-0112-82) and 0.5 µg of Rat IgG2a K Isotype Control, Super Bright 702 (Product # 67-4321-82) (left) or 0.5 µg of F4/80 Monoclonal Antibody, Super Bright 702 (right). Cells in the large scatter population were used for analysis.

IL12
BD Horizon™ V450 Dye is part of the BD Horizon™ violet family of dyes. This is a small organic fluorochrome with an excitation maximum (Ex Max) at 405-nm and an emission maximum (Em Max) at 450-nm. BD Horizon™ V450, driven by BD innovation, is designed to be excited by the violet laser (405 nm) and detected using an optical filter centered near 450-nm (e.g., a 450/50-nm bandpass filter). The dye can be excited by the UV (355-nm) laser resulting in cross-laser excitation and spillover. Please ensure that your instrument's configurations (lasers and optical filters) are appropriate for this dye.

IFNg
The XMG1.2 antibody is specific for mouse Interferon-gamma (IFN-g), a 20 kDa type II cytokine known for its central roles in protection against bacterial or viral pathogens and for its anti-tumor properties. IFN-g is secreted by several types of immune cells which allow the cytokine to modulate innate immunity when secreted by NK and NKT cells, and to function in support of adaptive immunity when secreted by Th1 and CD8+ T cells (CTLs).

IL10
Each lot of this antibody is quality control tested by intracellular immunofluorescent staining with flow cytometric analysis. For flow cytometric staining, the suggested use of this reagent is ≤0.125 µg per million cells in 100 µl volume. It is recommended that the reagent be titrated for optimal performance for each application.

TNFa
The BD Horizon Brilliant Violet™ 510 (BV510) Dye is part of the BD Horizon Brilliant Violet™ family of dyes. This polymer-technology based dye with an excitation maximum (Ex Max) at 327-nm / 405-nm and an emission maximum (Em Max) at 512-nm. BV510, driven by BD innovation, is designed to be excited by the violet laser (405-nm) and detected using an optical filter centered near 510-nm (e.g., a 525/50 bandpass filter). The dye can be excited by the UV (355-nm) laser resulting in cross-laser excitation and spillover. Please ensure that your instrument's configurations (lasers and optical filters) are appropriate for this dye.

CD11b
The M1/70 antibody reacts with human and mouse CD11b, also known as integrin αalpha M. This 165-170 kDa cell surface glycoprotein is part of a family of integrin αreceptors that mediate adhesion between ≥ ≥ ≥ cells (cell-cell) and components of the extracellular matrix, e.g. fibrinogen (cell-matrix). In addition, integrin αs are active signaling receptors which recruit leukocytes to inflammatory sites and promote cell activation. Complete, functional integrin αreceptors consist of distinct combinations of integrin αchains which are differentially expressed. integrin αalpha M (CD11b) assembles with integrin αbeta-2 (CD18) into a receptor known as Macrophage Antigen-1 (Mac-1) or complement receptor type 3 (CR3). This receptor binds and induces intracellular signaling through ICAM-1 on endothelial cells and can also facilitate removal of iC3b bearing foreign cells.The M1/70 antibody is widely used as

a marker for CD11b expression on mouse macrophages, granulocytes, neutrophils, and NK cells. The antibody is also reported to be cross-reactive for Rhesus macaque CD11b.
CD163
Description: This TNKUPJ monoclonal antibody recognizes mouse CD163. CD163 is a 130kDa surface receptor expressed by certain subsets of tissue macrophages, including splenic red pulp macrophages, Kupffer cells, intestinal lamina propria macrophages and a small fraction of peritoneal macrophages. In contrast to human blood monocytes, mouse monocytes do not express CD163. Also, unlike human CD163, mouse CD163 is not as readily induced by M2 polarizing cytokines, and it is not a good marker of M2 macrophages. No common cell lines of monocytic or macrophage origin express mouse CD163. In humans, CD163 has been shown to be proteolytically cleaved and shed from the cell surface, and it acts as a soluble anti-inflammatory factor.

CD206
This MR6F3 antibody has been reported for use in flow cytometric analysis, and intracellular staining followed by flow cytometric analysis.
Applications Tested: This MR6F3 antibody has been tested by intracellular staining and flow cytometric analysis of mouse resident peritoneal exudate cells using the Intracellular Fixation & Permeabilization Buffer Set (cat. 88-8824) and protocol. This can be used at less than or equal to 0.25 µg per test. A test is defined as the amount (µg) of antibody that will stain a cell sample in a final volume of 100 µL. Cell number should be determined empirically but can range from 10^5 to 10^8 cells/test. It is recommended that the antibody be carefully titrated for optimal performance in the assay of interest.

CD28 Antibody
This monoclonal antibody preparation was purified from tissue culture supernatant via affinity chromatography. For In Vivo Ready™ (IVR) products, each preparation is also evaluated for endotoxin levels using the LAL assay. It is recommended to store the product undiluted at 4°C. Do not freeze.

CD3 Antibody
CD3, also known as T3, is a member of the Ig superfamily and primarily expressed on T cells, NK-T cells, and at different levels on thymocytes during T cell differentiation. CD3 is composed of CD3ε, δ, γ and ζ chains. Quality tested for Flow cytometery.

Eukaryotic cell lines

Policy information about cell lines and Sex and Gender in Research

Cell line source(s)	B16F10 - ATCC and YUMM1.1 - generous gift from Dr. David Klinke, West Virginia University - original source Dr. Bosenberg, Yale University
Authentication	None of the cells were authenticated
Mycoplasma contamination	Cell lines were not tested Mycoplasma contamination
Commonly misidentified lines (See ICLAC register)	There are no commonly misidentified cell lines were used in the study

Animals and other research organisms

Policy information about studies involving animals; ARRIVE guidelines recommended for reporting animal research, and Sex and Gender in Research

Laboratory animals	C57BL/6J mice were used for the entire study.<br>1st murine model study – Female 6-8 week-old.<br>2nd murine model study – Female 6-8week-old.
Wild animals	No wild animals were used in the study.
Reporting on sex	Sex as a variable was not tested in these studies.
Field-collected samples	No field collected samples were used in the study.
Ethics oversight	IACUC, Arizona State University

Note that full information on the approval of the study protocol must also be provided in the manuscript.

Flow Cytometry

Plots

Confirm that:

☒ The axis labels state the marker and fluorochrome used (e.g. CD4-FITC).

☒ The axis scales are clearly visible. Include numbers along axes only for bottom left plot of group (a 'group' is an analysis of identical markers).

☒ All plots are contour plots with outliers or pseudocolor plots.

☒ A numerical value for number of cells or percentage (with statistics) is provided.

Methodology

Sample preparation	Flow cytometry (FACS) staining buffer was prepared by generating 1% bovine serum albumin (VWR, Radnor, PA), 2 mM Na2EDTA (VWR, Radnor, PA) and 0.1% NaN3 (VWR, Radnor, PA). Live/dead staining was performed using fixable dye eF780 (ThermoFisher Scientific, Waltham, MA, USA). All antibodies required for staining were purchased and used as is (BD biosciences, Tonbo Biosciences, BioLegend, Thermo Scientific, Invitrogen).
Instrument	Flow cytometry was performed by following the manufacturer's recommendation and guidelines set by ASU flow cytometry core using Attune NXT Flow cytometer (ThermoFisher Scientific, Waltham, MA, USA).
Software	FlowJo software was used to analyze the FCS files obtained from Attune NXT Flow cytometer
Cell population abundance	A minimum of 40,000 total events were recorded for all samples. Minimum cell sensitivity of the instrument was considered for all relevant cell populations within post-sort fractions.
Gating strategy	A preliminary FSC-H v/s SSC-H gating was used for all cells. In this sub population, single cell population (FSC-A v/s FSC-H) was gated. All relevant sub-populations were further gated within the single cell population. Comp beads were used to determine the “positive” and “negative” staining cell populations. Specifically, the start of the 2nd peak (positive peak) was determined to be positive boundary for the cell populations.

☒ Tick this box to confirm that a figure exemplifying the gating strategy is provided in the Supplementary Information.
